# Osteopontin Deficiency Suppresses Intestinal Tumor Development in *Apc*-Deficient Min Mice

**DOI:** 10.3390/ijms18051058

**Published:** 2017-05-14

**Authors:** Rikako Ishigamori, Masami Komiya, Shinji Takasu, Michihiro Mutoh, Toshio Imai, Mami Takahashi

**Affiliations:** 1 Central Animal Division, National Cancer Center Research Institute, 5-1-1, Tsukiji, Chuo-ku, Tokyo 104-0045, Japan; rishigam@ncc.go.jp (R.I.); toimai@ncc.go.jp (T.I.); 2 Epidemiology and Prevention Division, Research Center for Cancer Prevention and Screening, National Cancer Center, 5-1-1, Tsukiji, Chuo-ku, Tokyo 104-0045, Japan; mkomiya@ncc.go.jp (M.K.); mimutoh@ncc.go.jp (M.M.); 3 Division of Pathology, National Institute of Health Science, 1-18-1 Kamiyoga, Setagaya-ku, Tokyo 158-8501, Japan; s-takasu@nihs.go.jp

**Keywords:** osteopontin, colorectal tumor, macrophage

## Abstract

Osteopontin (OPN) is a secreted phosphoglycoprotein, and is a transcriptional target of aberrant Wnt signaling. OPN is upregulated in human colon cancers, and is suggested to enhance cancer progression. In this study, the effect of deficiency of OPN on intestinal tumor development in *Apc*-deficient Min mice was investigated. At 16 weeks of age, the number of small intestinal polyps in Min/OPN(+/−) and Min/OPN(−/−) mice was lower than that of Min/OPN(+/+) mice. Colorectal tumor incidences and multiplicities in Min/OPN(+/−) and Min/OPN(−/−) mice were significantly lower than those in Min/OPN(+/+) mice, being 48% and 0.6 ± 0.8, 50% and 0.8 ± 0.9 vs. 80% and 1.6 ± 1.7, respectively. OPN expression in colorectal tumors was strongly upregulated in Min/OPN(+/+) compared to adjacent non-tumor parts, but was decreased in Min/OPN(+/−) and not detected in Min/OPN(−/−). Targets of OPN, matrix metalloproteinases (MMPs)-3, -9, and -13 were lowered by OPN deficiency. Macrophage marker F4/80 in colorectal tumors was also lowered by OPN deficiency. MMP-9 expression was observed in tumor cells and tumor-infiltrating neutrophils. These results indicate that induction of OPN by aberrant Wnt signaling could enhance colorectal tumor development in part by upregulation of MMP-3, -9, and -13 and infiltration of macrophage and neutrophils. Suppression of OPN expression could contribute to tumor prevention, but complete deficiency of OPN may cause some adverse effects.

## 1. Introduction

Osteopontin (OPN), also known as secreted phosphoprotein 1 (SPP1), binds to several integrin receptors including CD44v6, a splicing variant of CD44, which is a marker of colon cancer stem cells, and regulates cell motility, invasion, chemotaxis, and cell survival [1,2]. OPN is overexpressed in multiple types of cancer, including colorectal carcinomas [3,4], and serum levels of OPN in cancer patients are elevated. Thus, it is used as a diagnostic and prognostic marker [5]. OPN plays important roles in immune regulation [6,7,8] and cancer progression [9,10]. OPN expression in colon cancer has been identified as an independent prognostic parameter for overall survival, and high OPN expression is associated with bad prognosis [11]. This might be explained by OPN being implicated as a key regulatory component of epithelial-mesenchymal transition (EMT) [12]. OPN is expressed in tumor cells and tumor-associated macrophages (TAMs) [13], and both autocrine and paracrine signaling of OPN are considered to be involved in tumor progression. Indeed, it has been reported that both endogenous OPN expression and exogenous OPN enhances the motility and invasiveness of human colon cancer cells in vitro [14]. OPN enhances hepatic metastasis of colorectal cancer cells [15], and it has been reported that silencing of OPN by small interfering RNA (siRNA) suppresses murine colon adenocarcinoma metastasis [16]. OPN knockdown in a human colon carcinoma cell line by siRNA reduces vascular endothelial growth factor (VEGF), matrix metalloproteinase (MMP)-2, and MMP9, and suppresses colon cancer cell growth [17]. OPN is also abundant in bone, and facilitates bone metastasis of breast cancer [18]. Thus, OPN is considered to be a candidate target for cancer therapy [19,20]. However, it is not clear whether OPN could be a target for cancer prevention.

Epidemiological studies have shown that insulin resistance and obesity are risk factors for colorectal tumors [21,22]. OPN is upregulated in adipose tissue in obesity and causes adipocyte inflammation and insulin resistance through macrophage activation [23,24]. Deficiency of OPN prevents proliferation of macrophages in adipose tissue, and induction of insulin resistance and inflammation in adipose tissue induced by a high fat diet in mice [23,25,26]. Neutralization of OPN by anti-OPN antibody also inhibits obesity-induced inflammation and insulin resistance in diet-induced obese mice [27]. Increased circulating levels of OPN have been observed to be due to obesity and colon cancer [28]. Thus, suppression of circulating OPN levels could prevent colorectal tumor development. There are reports that OPN depletion inhibits diethylnitrosamine (DEN)-induced hepatocarcinogenesis and *N*-methyl-*N*-nitrosourea (MNU) and *Helicobacter pylori*-induced gastric cancer development in mice [29,30]. However, there are no reports about intestinal tumorigenesis in *OPN*-knockout mice.

In human colon cancers, the *Apc* gene, a gene responsible for familial adenomatous polyposis (FAP), is frequently mutated [31] and Wnt/beta-catenin signaling is aberrantly activated [32]. OPN has been suggested to be a putative target of Wnt signaling, and elevated expression of OPN has been reported to be significantly correlated with increased cytoplasmic and nuclear accumulation of beta-catenin [11]. In genetically defined mouse models, OPN is upregulated in tumors in *Apc^1638N^* mice, an *Apc*-deficient mouse model, but not in tumors in *pvillin-KRAS^V12G^* mice without Wnt activation mutations [11]. The Min mouse, another animal model of FAP, harbors a mutation and develops numerous polyps in the intestinal tract [33]. In the present study, the effect of deficiency of OPN on intestinal tumor development in *Apc*-deficient Min mice was investigated to clarify the importance of OPN in the early phase of colon tumor development.

## 2. Results

### 2.1. Effect of Osteopontin (OPN) Deficiency on Intestinal Polyp Formation in Min Mice

To investigate involvement of OPN in intestinal tumor development, the effect of the deficiency of OPN on intestinal polyp formation in Min mice was examined. *OPN* genotypes did not significantly affect food intake, behavior, or body weight changes during the experimental periods. Final body weights (g) in male Min/OPN(+/+), Min/OPN(+/−), Min/OPN(−/−), OPN(+/+), OPN(+/−), and OPN(−/−) mice were 23.3 ± 5.3, 26.0 ± 4.5, 25.2 ± 4.5, 29.8 ± 2.6, 31.4 ± 2.0, and 31.8 ± 2.1, respectively (Figure S1a). The differences between *Apc* mutant and wild type mice were statistically significant (*p* < 0.05). Final body weights (g) in female Min/OPN(+/+), Min/OPN(+/−), Min/OPN(−/−), OPN(+/+), OPN(+/−), and OPN(−/−) mice were 17.9 ± 3.3, 20.5 ± 2.1, 19.9 ± 2.3, 22.1 ± 1.1, 21.7 ± 1.9, and 22.3 ± 1.9, respectively (Figure S1b). The differences between male and female mice of each genotype were statistically significant (*p* < 0.01). There were no significant differences in final body weights among OPN genotypes. On the other hand, OPN genotypes affected spleen weights of *Apc* mutant mice. Spleen weights (g) in male Min/OPN(+/+), Min/OPN(+/−), Min/OPN(−/−), OPN(+/+), OPN(+/−), and OPN(−/−) mice were 0.173 ± 0.078, 0.278 ± 0.181, 0.270 ± 0.128, 0.092 ± 0.029, 0.105 ± 0.050, and 0.096 ± 0.021, respectively (Figure S1c). Spleen weights (g) in female Min/OPN(+/+), Min/OPN(+/−), Min/OPN(−/−), OPN(+/+), OPN(+/−), and OPN(−/−) mice were 0.156 ± 0.086, 0.174 ± 0.086, 0.232 ± 0.156, 0.088 ± 0.010, 0.088 ± 0.016, and 0.093 ± 0.027, respectively (Figure S1d). Spleen weights of *Apc* mutant mice were higher than those of Apc(+/+) mice, and deficiency of OPN further increased the spleen weight. OPN genotypes did not affect spleen weights of mice without the *Apc* mutation.

**Figure 1:**
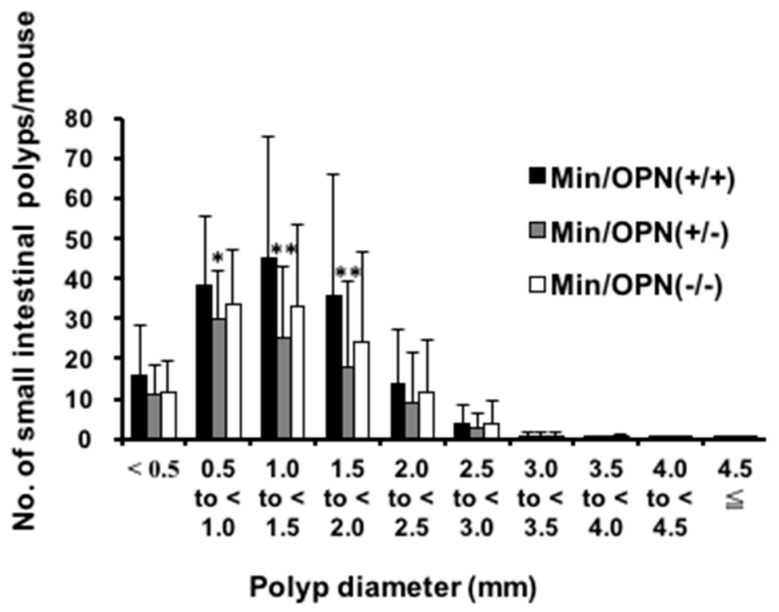
The effect of OPN deficiency on the size distribution of small intestinal polyps in Min mice. The number of polyps per mouse in each size class is given as a mean. Significant difference from Min/OPN(+/+) mice (* *p* < 0.05, ** *p* < 0.01).

Table 1 summarizes the data for the number and distribution of small intestinal polyps in the Min/OPN(+/+), Min/OPN(+/−), and Min/OPN(−/−) mice at 16 weeks of age. Most polyps developed in the middle and distal sections of the small intestine, with only a few in the proximal segment of the small intestine and in the colon. There were no polyps in OPN(+/+), OPN(+/−), and OPN(−/−) mice. The total numbers of small intestinal polyps in Min/OPN(+/−) (96.3 ± 57.4, *p* < 0.01) and Min/OPN(−/−) mice (117.1 ± 62.4) were lower than that of Min/OPN(+/+) mice (152.8 ± 93.6). The majority of polyps were observed in the size range between 0.5 and 2.0 mm in diameter (Figure 1). In comparison to Min/OPN(+/+) mice, the number of polyps in the size range between 0.5 and 2.0 mm in diameter remarkably decreased in Min/OPN(+/−) and Min/OPN(−/−) mice.

**Figure 2:**
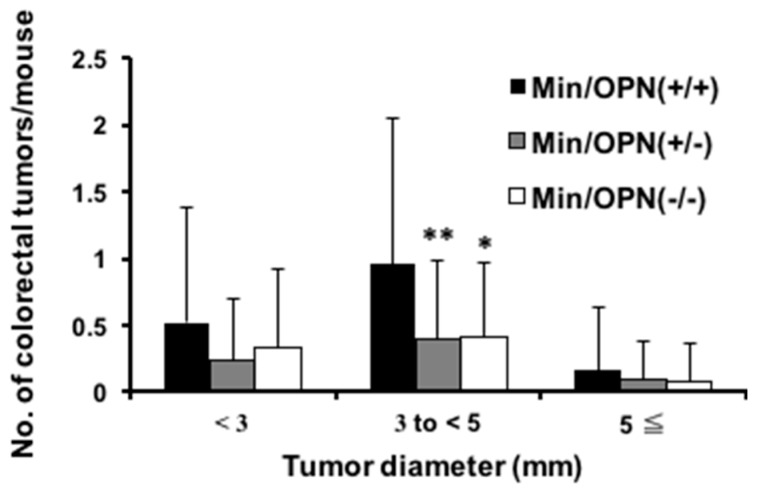
The effect of OPN deficiency on size distribution of colon tumors in Min mice. The number of tumors per mouse in each size class is given as a mean. Significant difference from Min/OPN(+/+) mice (* *p* < 0.05, ** *p* < 0.01).

As shown in Figure S2, colorectal tumors developed in the male and female Min/OPN(+/+), Min/OPN(+/−), and Min/OPN(−/−) mice. No lesions were observed in mice without the *Apc* gene mutation. Data for the incidence and multiplicity of colon tumors are summarized in Table 2. Both colon tumor incidences and multiplicities in Min/OPN(+/−) and Min/OPN(−/−) mice were significantly lower than those in Min/OPN(+/+) mice, being 27/56 (48%) (*p* < 0.01) and 0.6 ± 0.8 (*p* < 0.01), 18/36 (50%) (*p* < 0.05) and 0.8 ± 0.9 (*p* < 0.01) vs. 20/25 (80%) and 1.6 ± 1.7, respectively. Histopathological examination revealed that incidences of adenomas and adenocarcinomas in Min/OPN(+/+) mice were 44% and 60%, respectively, and each incidence tended to decrease with genetic OPN deficiency. Moreover, multiplicities of adenoma and adenocarcinoma also tended to decrease by OPN deficiency. Figure 2 shows the size distribution of colorectal tumors in mice. Compared to Min mice, the number of tumors showed a tendency to decrease at all sizes in mice with OPN deficiency. The number of tumors ranging between 3.0 mm and 5.0 mm in diameter was statistically lower in Min/OPN(+/−) (0.4 ± 0.6, *p* < 0.01) and Min/OPN(−/−) (0.4 ± 0.6, *p* < 0.05) mice than that in Min/OPN(+/+) mice (1.0 ± 1.1).

### 2.2. Serum Levels *of OPN, Interleukin (IL)-6, and Triglycerides*

The serum levels of OPN in Min/OPN(+/+), Min/OPN(+/−), and Min/OPN(−/−) mice were significantly different from each other and OPN genotype dependent (Table 3). As for the OPN(+/+), OPN(+/−), and OPN(−/−) mice, the OPN levels were also significantly different from each other and OPN genotype dependent. The serum levels of OPN in Min/OPN(+/+) were slightly higher than in OPN(+/+) mice, though the difference was not statistically different. The serum levels of OPN in Min/OPN(+/−) were significantly higher than in OPN(+/−) mice.

The serum levels of IL-6 were significantly elevated in mice bearing the *Apc* gene mutation (Min/OPN(+/+), Min/OPN(+/−), and Min/OPN(−/−)) compared with those in mice without the *Apc* gene mutation (OPN(+/+), OPN(+/−), and OPN(−/−) ), respectively (Table 3). The serum IL-6 level in Min/OPN(−/−) mice was significantly lower than those in Min/OPN(+/+) and Min/OPN(+/−) mice.

Mice bearing the *Apc* gene mutation were in the hypertriglyceridemic state, as shown in Table 3. In Min/OPN(+/−) and Min/OPN(−/−) mice, the levels of triglycerides (TGs) were lower than that in Min/OPN(+/+) mice, though the differences were not statistically significant. On the other hand, the TG levels in mice without the *Apc* gene mutation were low and similar among OPN(+/+), OPN(+/−), and OPN(−/−) mice. The serum levels of TGs in mice bearing the *Apc* gene mutation were statistically higher than that in mice without the *Apc* gene mutation (*p* < 0.01).

### 2.3. Correlation of Small Intestinal Polyp Numbers with Serum Levels *of Triglycerides and Spleen Weights*

**Figure 3:**
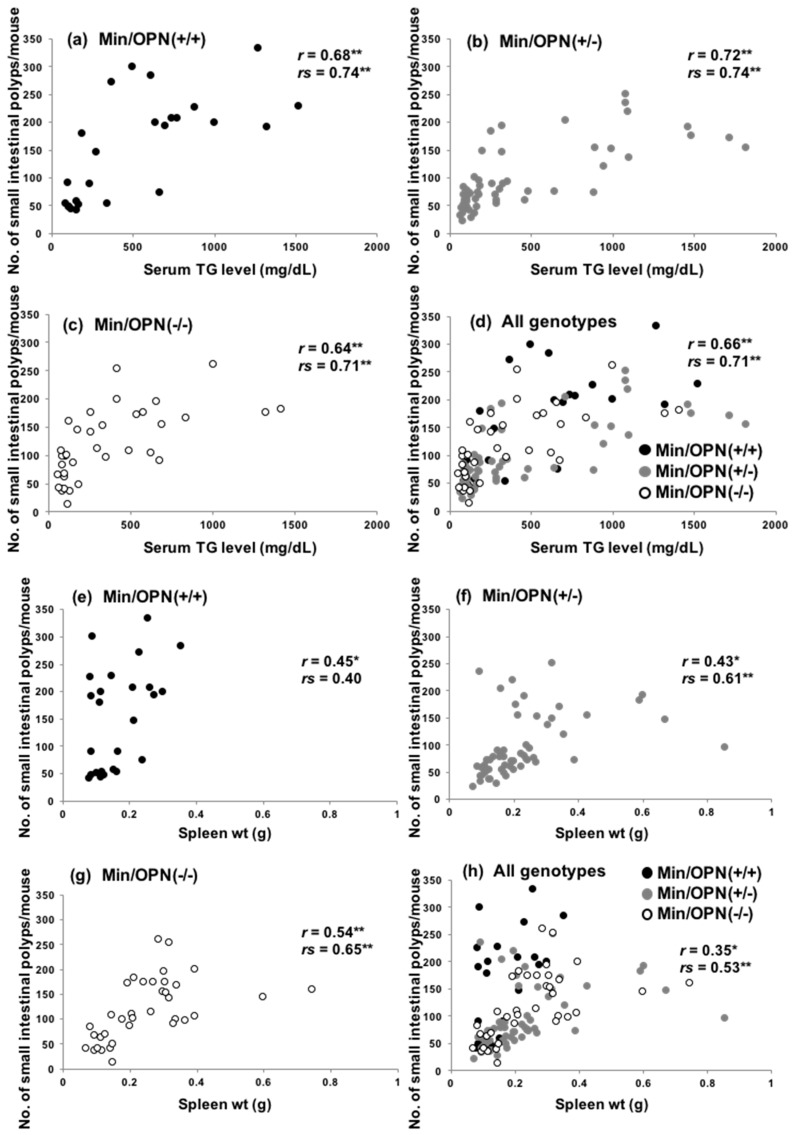
Scatter plots of serum triglyceride (TG) levels and intestinal polyp number in (**a**) Min/OPN(+/+), (**b**) Min/OPN(+/−), and (**c**) Min/OPN(−/−) mice, and (**d**) all genotypes. Scatter plots of spleen weight and intestinal polyp number in (**e**) Min/OPN(+/+), (**f**) Min/OPN(+/−), and (**g**) Min/OPN(−/−) mice, and (**h**) all genotypes. *r*, the Pearson correlation coefficient; *rs*, Spearman’s rank correlation coefficient; *, **, the correlation was statistically significant at *p* < 0.005, and *p* < 0.0005, respectively.

Previously, we reported that serum TG levels dramatically increase with age in Apc-deficient mice, including Min mice, and both hyperlipidemia and polyp formation were suppressed by administration of peroxisome proliferator-activated receptor (PPAR) γ ligands, suggesting that hyperlipidemia in Min mice may be associated with intestinal lesion development [34]. Accordingly, the TG levels and number of small intestinal polyps in each mouse in the present study were plotted. As shown in Figure 3a–d, significant positive correlation between serum levels of TGs and polyp numbers was observed in Min/OPN(+/+) (*r* = 0.68 by the Pearson correlation coefficient test, *p* = 0.00019; *rs* = 0.74 by Spearman’s rank correlation coefficient test, *p* = 0.00031) Min/OPN(+/−) (*r* = 0.72, *p* = 3.1 × 10^−10^; *rs* = 0.74, *p* = 3.4 × 10^−8^), Min/OPN(−/−), (*r* = 0.64, *p* = 3.0 × 10^−5^; *rs* = 0.71, *p* = 2.5 × 10^−5^), and all three genotypes (*r* = 0.66, *p* = 9.5 × 10^−16^; *rs* = 0.71, *p* = 2.7 × 10^−15^).

It has been reported that spleen weights in Min mice positively correlate with small intestinal polyp numbers [35,36]. However, spleen weights of OPN-deficient Min mice were heavier than those of Min/OPN(+/+) mice in the present study (Figure S1c,d). The correlation between spleen weights and small intestinal polyps was examined (Figure 3e–h). Positive correlations were observed in Min/OPN(+/+) (*r* = 0.45 by the Pearson correlation coefficient test, *p* = 0.024; *rs* = 0.40 by Spearman’s rank correlation coefficient test, *p* = 0.048), Min/OPN(+/−) (*r* = 0.43, *p* = 0.0011; *rs* = 0.61, *p* = 5.2 × 10^−6^), Min/OPN(−/−) (*r* = 0.54, *p* = 0.00065; *rs* = 0.65, *p* = 00011), and all three genotypes (*r* = 0.35, *p* = 0.00013; *rs* = 0.53 *p* = 9.9 × 10^−9^), though the correlation coefficients were not very high. In the Min/OPN(+/+) group, there were no mice with more than 0.4 g of spleen weight, even though there were mice with quite high polyp numbers (250 <). On the other hand, in the Min/OPN(+/−) and Min/OPN(−/−) groups, there were a few mice with quite high spleen weights, though their polyp numbers were not very high (between 100 and 200); that is to say, positive correlations between polyp numbers and spleen weights were observed in each genotype, but the gradient of the correlation line seems to be different between Min/OPN(+/+) and OPN-deficient Min mice. As shown in Figure 1 and Figure 2, numbers of large polyps were relatively low in Min/OPN(+/−) and Min/OPN(−/−) mice compared with those in Min/OPN(+/+) mice.

### 2.4. Effects of OPN deficiency on Gene Expression Levels in Colon Tumors

**Figure 4:**
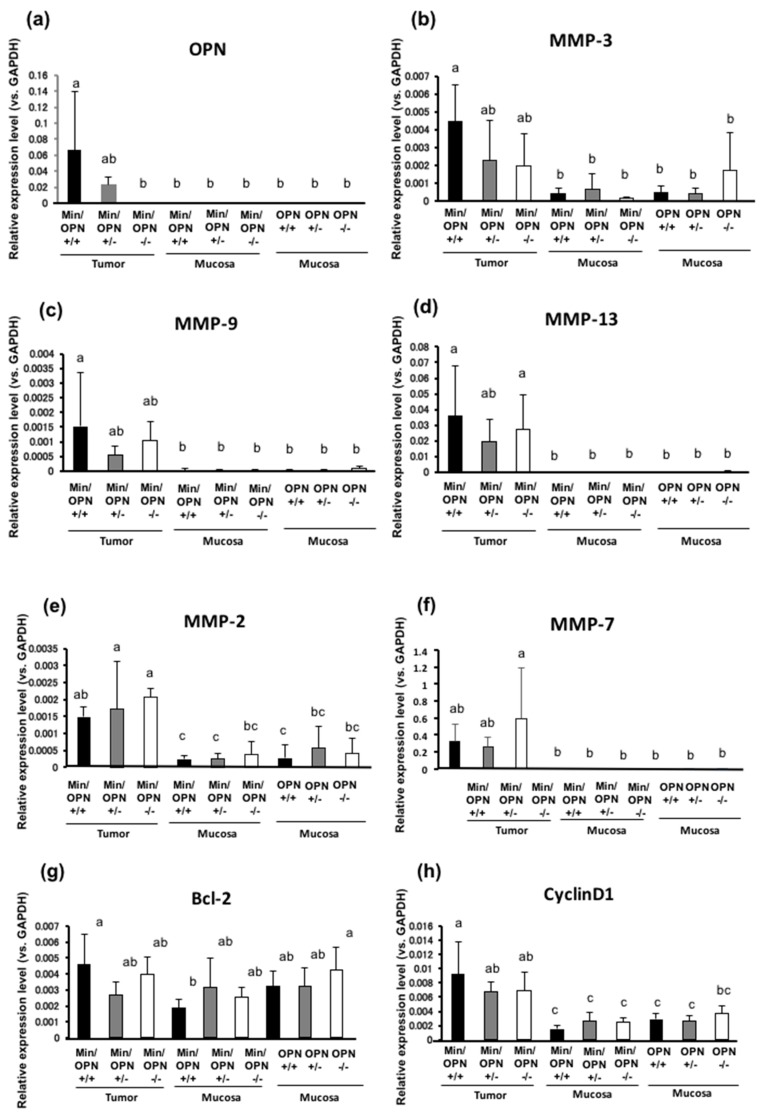

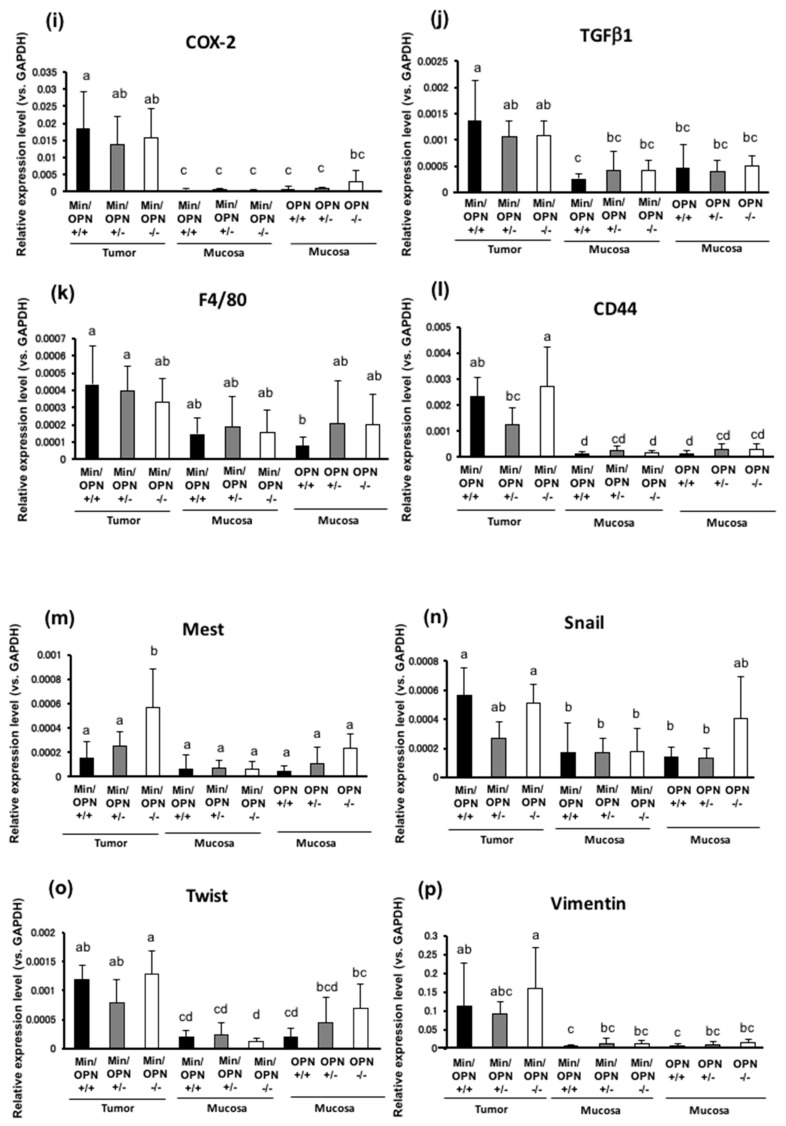
Effects of OPN deficiency on mRNA expression levels. Values for relative mRNA expression levels (vs. glyceraldehyde 3-phosphate dehydrogenase (GAPDH) of (**a**) OPN, (**b**) matrix metalloproteinase (MMP)-3, (**c**) MMP-9, (**d**) MMP-13, (**e**) MMP-2, (**f**) MMP-7, (**g**) Bcl-2, (**h**) CyclinD1, (**i**) COX-2, (**j**) transforming growth factor (TGF) β1, (**k**) F4/80, (**l**) CD44, (**m**) Mest, (**n**) Snail, (**o**) Twist, and (**p**) Vimentin. Data are means ± SD. Values that do not share a common superscript are significantly different at *p* < 0.05.

The effects of OPN deficiency on gene expression levels in colorectal tumors and non-tumorous colorectum were investigated by semi-quantitative reverse transcription-polymerase chain reaction (RT-PCR) analysis. OPN expression in colorectal tumors was strongly upregulated in Min/OPN(+/+) compared to the adjacent non-tumor part. These *OPN* levels were decreased in Min/OPN(+/−) and not detected in Min/OPN(−/−) (Figure 4a). OPN has been reported to activate MMPs. MMP-3, MMP-9, MMP-13, MMP-2, and MMP-7 were upregulated in colorectal tumors in Min/OPN(+/+) compared to adjacent non-tumor parts. The elevated expression levels of MMP-3 were decreased to almost half by hetero-knockout of *OPN*, and further decreased by homo-knockout (Figure 4b). The elevated expression levels of MMP-9 and MMP-13 were decreased to almost half by hetero-knockout of *OPN*, while a decrease by homo-knockout of *OPN* was slight and not significant (Figure 4c,d). On the other hand, MMP-2 and MMP-7 expression levels in the colorectal tumors were further increased by OPN deficiency (Figure 4e,f). Expression levels of cell survival/growth-related genes, Bcl-2, CyclinD1, COX-2, and transforming growth factor (TGF) β1 were higher in colorectal tumors than those in adjacent colorectal mucosa in Min/OPN(+/+) mice. Those expression levels in the colorectal tumors were slightly decreased in Min/OPN(+/−) and Min/OPN(−/−) mice (Figure 4g–j). Expression of a macrophage marker F4/80 in colorectal tumors was also slightly lowered in Min/OPN(+/−) and Min/OPN(−/−) mice (Figure 4k). CD44, a target of Wnt signaling [37] and a receptor of OPN, was upregulated in tumors in Min/OPN(+/+) mice, and the expression was decreased to almost half by hetero-knockout of *OPN*, but not by homo-knockout (Figure 4l). Interestingly, expression of mesoderm-specific transcript (Mest)/paternally expressed gene 1 (Peg1), an inhibitory factor of Wnt signaling [38], was inversely associated with OPN dose in tumors, and it was significantly elevated in tumors compared with adjacent non-tumor parts in Min/OPN(−/−) mice (Figure 4m). Expression levels of EMT-related genes, Snail and Twist, were higher in colorectal tumors than those in adjacent colorectal mucosa in Min/OPN(+/+) mice, and those were decreased in Min/OPN(+/−) mice, but not in Min/OPN(−/−) mice (Figure 4n,o). Vimentin expression was also upregulated in tumors in Min/OPN(+/+) mice, and further increased in Min/OPN(−/−) mice (Figure 4p).

### 2.5. Protein Expression in Colon Tumors

**Figure 5:**
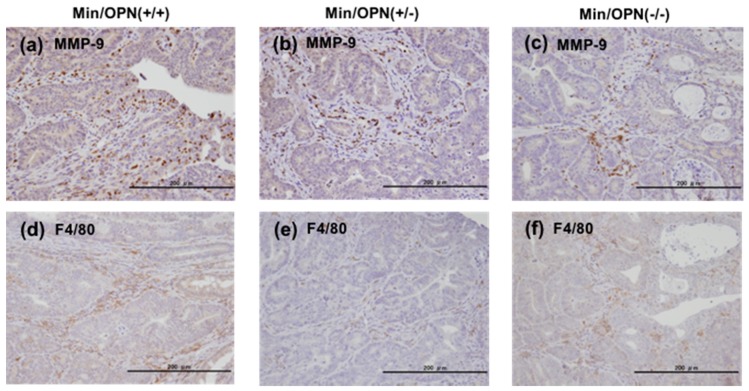
Protein expression in colorectal tumor tissue. (**a**–**c**) MMP-9 and (**d**–**f**) macrophage marker F4/80 were immunohistochemically stained in colorectal tumors in Min/OPN(+/+), Min/OPN(+/−), and Min/OPN(−/−) mice, respectively. Objective magnification: ×40.

Protein expressions in colorectal tumors in Min/OPN(+/+), Min/OPN(+/−), and Min/OPN(−/−) mice were examined by immunohistochemical staining. MMP-9 expression was observed strongly in stromal infiltrating neutrophils, and weakly in cancer cells in tumor tissue in Min/OPN(+/+) mice (Figure 5a). Lower expression of MMP-9 was observed in tumor tissue in Min/OPN(+/−) and Min/OPN(−/−) mice (Figure 5b,c). F4/80-positive macrophages were observed to be accumulated in tumor stroma in Min/OPN(+/+) mice (Figure 5d), and lower numbers of macrophages were observed in Min/OPN(+/−) and Min/OPN(−/−) mice (Figure 5e,f).

## 3. Discussion

In the present study, OPN-deficient Min mice showed decline in the number and size of small intestinal polyps compared to those of Min/OPN(+/+) mice in both males and females at the age of 16 weeks. Furthermore, OPN-deficient Min mice exhibited decreased incidence, multiplicity, and size of colorectal tumors. OPN expression was markedly elevated in colorectal tumors compared with that in adjacent normal colon mucosa in Min/OPN(+/+) mice, and that decreased with the *OPN* gene dosage. Elevated expressions of MMP-3, MMP-9, and MMP-13 in colorectal tumors in Min/OPN(+/+) mice were decreased by OPN deficiency. MMP-9 expression was observed in tumor cells and tumor-infiltrating neutrophils in Min/OPN(+/+) mice. Macrophage marker F4/80 in colorectal tumors was also lowered by OPN deficiency. These results indicate that OPN could enhance tumorigenesis in part by upregulating MMPs and increasing tumor-infiltrating neutrophils and macrophages, and could be a target for cancer prevention.

In Min mice, it has been reported that heterozygous disruption of the phosphatase and tensin homolog (PTEN) strongly induces OPN expression and promotes intestinal neoplasia [39]. Knockdown of OPN expression in human colon cancer cells suppresses cell proliferation, adherence, invasion, and expression of angiogenetic factors, such as VEGF, MMP-2, and MMP-9 [17]. It has been reported that tumor-infiltrating MMP-9-positive neutrophils enhance angiogenesis [40]. OPN is involved in neutrophil infiltration [41], and neutralization of OPN attenuates neutrophil migration [42]. OPN activates the phosphoinositide 3-kinase (PI3K)-phospo-Akt-nuclear factor (NF)-κB signaling pathway via α(v)β(3) integrin binding [43]. OPN promotes expression of MMP-13 through NF-κB signaling in osteoarthritis [44]. MMP-3 and MMP-13 is upregulated in human colorectal carcinomas [45], and MMP-13 activity is associated with poor prognosis in colorectal cancer [46]. OPN signaling also upregulates COX-2 expression via α(9)β(1) integrin [47]. Moreover, it has been reported that OPN activates JAK2/STAT3 signaling and upregulates Bcl-2 and cyclinD1 in human breast cancer cells [48]. OPN activates macrophages [13] and modulates EMT [12]. Consistent with these reports, elevated expression levels of MMP-3, MMP-9, and MMP-13 were lowered by OPN deficiency in the present study. Elevated expression levels of Bcl-2, CyclinD1, COX-2, TGF β1, and F4/80 in colorectal tumors in Min mice were only slightly lowered by OPN deficiency. Since Cyclin D1 and COX-2 are also known to be targets of β-catenin/Lef-1 [49,50], the effects of OPN knockout would be relatively small. On the other hand, elevated expressions of MMP-2 and MMP-7 in colorectal tumors in Min mice were not lowered but rather increased by OPN deficiency in the present study. Elevated expression of CD44 and EMT-related genes, Snail and Twist in tumors in Min/OPN(+/+) were lowered by OPN hetero-deficiency, but not by homo-deficiency. The reasons for this are uncertain. In the present study, we found that Mest, which has been reported to be an inhibitory factor of Wnt signaling [38] and has been upregulated in obese adipose tissue [51], was significantly elevated in colorectal tumors of Min/OPN(−/−) mice. It has been reported that leptin, an obesity-related factor, upregulates MMP-2 [52] and induces EMT [53]. As a bone marker, OPN is inversely associated with leptin in non-diabetic women [54]. Though the roles of Mest in tumorigenesis are unknown, we speculate that it may affect tumorigenesis via upregulation of MMPs and EMT-related genes in tumors in Min/OPN(−/−). We are now investigating the roles of Mest in tumorigenesis.

OPN is a secreted protein. The serum OPN levels in the Min/OPN(+/−) mice were almost half compared to those in Min/OPN(+/+) mice. Serum OPN was not detected in Min/OPN(−/−) mice. These results are consistent with the *OPN* gene dosage. The differences of serum OPN levels between mice with and without the *Apc* mutation, which are considered to be due to OPN production by intestinal tumors, were not particularly marked. This means that contribution of OPN produced in the tumors to the circulation levels of OPN was not high in Min mice. Min mice develop many polyps in the small intestine, but most of them are adenomas. Some colorectal tumors are carcinomas, but tumor volumes are relatively small. Besides cancer, OPN is expressed in a variety of tissues and cells including adipocytes and macrophages, and highly upregulated in inflammation [7]. This circulating OPN could also contribute to tumor development.

In the present study, serum IL-6 levels were elevated in mice bearing the *Apc* gene mutation, and that was lowered by homo-deficiency of OPN. Serum IL-6 levels positively correlate with progression of human colorectal cancer [55]. It has been reported that Min mice have elevated levels of circulating IL-6, which are decreased by exercise [36]. Min mice suffer from lymphodepletion between 83 and 120 days of age [56], and lymphodepletion could be associated with increased plasma IL-6 [57].

Epidemiological studies have shown that high serum TG levels are related with the risk of colorectal cancer [58,59]. Dysregulation of lipoprotein lipase (LPL) contributes to dyslipidemia, and LPL inducers, such as PPAR ligands, NO-1886, and indomethacin, have been shown to decrease TG levels and suppress tumor development in animal models [60]. Correlation between the level of TGs and the number of intestinal polyps was observed in the present study. In the OPN-deficient Min mice, serum TG levels tended to decrease with the *OPN*-gene dosage. It has been reported that osteogenic differentiation gene *OPN* and adipogenic differentiation gene *LPL* are oppositely regulated in mesenchymal stem cells [61,62]. These findings indicate that the depletion of OPN could affect development of small intestinal polyps and colorectal tumors in part through decreasing the inflammatory status and hypertriglyceridemia.

It has been reported that OPN is involved in high fat-induced insulin resistance and OPN deficiency protects against insulin resistance [22]. Therefore, insulin levels in the mice used in the present study were measured, but statistically significant differences were not observed (data not shown).

Intestinal polyposis causes anemia in Min mice [63], and as a result, extramedullary hematopoiesis in the spleen occurs [64,65]. Therefore, spleen weights in Min mice positively correlate with intestinal polyp numbers. Contrary to this, OPN deficiency increased spleen weight without an increase in polyp numbers and size in the present study. The reason is unclear. It has been reported that myocardial angiogenic response is impaired in the absence of OPN [66]. To recover the anemia, aggressive extramedullary hematopoiesis may play some roles in OPN-deficient Min mice.

Chronic inflammation is known to be a risk factor for cancer. *Helicobacter pylori* infection, which causes chronic gastritis, is closely associated with gastric cancer risk [67,68]. OPN depletion decreases inflammation and gastric epithelial proliferation during *Helicobacter pylori* infection in mice [69], and suppresses MNU and *Helicobacter pylori*-induced gastric cancer development [29]. As for colorectal cancer, inflammatory bowel diseases (IBDs), including ulcerative colitis (UC) and Crohn’s disease (CD), are well-known risk factors [70,71,72]. Increased levels of circulating and colonic tissue OPN in human IBD and experimentally-induced colitis in mice have been observed [73,74,75,76,77,78]. However, results of experimental studies about the effects of OPN deficiency on dextran sulfate sodium (DSS)-induced colitis are controversial. It has been reported that OPN deficiency exacerbates tissue destruction in DSS-induced acute colitis [74,79], and another report has shown that OPN deficiency protects mice from DSS-induced colitis [80]. In contrast to acute colitis, OPN-null mice are protected from mucosal inflammation during chronic colitis [74]. These findings suggest that OPN is a two-sided mediator of intestinal inflammation [74] and participates in both inflammation and mucosal protection in IBDs [73]. Thus, effects of OPN deficiency on colitis-associated colorectal carcinogenesis are unclear, and it is considered that suppression of mucosal protective effects of OPN may enhance colitis-associated colorectal carcinogenesis. OPN could be a target for tumor prevention under weak and chronic inflammation, such as in obesity, but when there are severe injury and acute inflammation, complete depletion of OPN should not be recommended. In the present study, suppressive effects of hetero-deficiency of OPN on intestinal tumor formation in Min mice were slightly higher than those of homo-deficiency. Since OPN plays important roles in many tissues and cells, complete suppression may cause adverse effects. Moreover, we speculate that Mest, which was found to be elevated in tumors in Min/OPN(−/−) mice, may affect tumorigenesis. It has been reported that OPN deficiency is linked to a reduced immune response [8]. Post-transcriptional activation of OPN by MMPs could also affect OPN functions. These points may have roles to play in the differential response. Roles of OPN in early stages of colorectal tumorigenesis and ways to prevent colorectal cancer development via OPN suppression should be further investigated.

## 4. Materials and Methods

### 4.1. Animals and Diets

Male and female C57BL/6-*Apc^Min/+^* mice (Min mice) and B6.129S6(Cg)-Spp1^tmlBlh^/J (JR#004936) (OPN(−/−) mice) (those were backcrossed to background C57BL/6 for 10 generations) were purchased from Jackson Laboratories (Bar Harbor, ME, USA). Min mice were mated with OPN(−/−) mice to generate Min/OPN(+/−) mice. Then, the Min/OPN(+/−) mice were crossed with OPN(+/−) mice to obtain Min/OPN(+/+), Min/OPN(+/−), Min/OPN(−/−), OPN(+/+), OPN(+/−), and OPN(−/−) as littermates. Since Apc-homo-deficient mice are embryonic lethal, all Min/OPN(+/+), Min/OPN(+/−), and Min/OPN(−/−) mice are Apc hetero-deficient. Offspring were genotyped by PCR as previously reported [33,81]. All mice were housed in plastic cages with sterilized softwood chips as bedding in a barrier-sustained animal room with controlled conditions of humidity (55%), light (12/12 h light/dark cycle), and temperature (24 ± 2 °C). Basal diet AIN-76A and water were available ad libitum. The animals were observed daily for clinical signs and mortality. The experiments were performed according to the “Guidelines for Animal Experiments of the National Cancer Center” and were approved by the Institutional Ethics Review Committee for Animal Experimentation of the National Cancer Center (permission code: T07-012, approval date: 1 April 2007). Diluted isoflurane [82] was used to anaesthetize the animals.

### 4.2. Analysis of Intestinal Polyps

At 16 weeks old, mice were anesthetized, and blood samples were collected from the abdominal vein. The intestinal tract was removed and separated into the small intestine, cecum, and colon. The small intestine was divided into the proximal segment (4 cm in length) and the proximal (middle) and distal halves of the remainder. These segments were opened longitudinally and fixed flat between sheets of filter paper in 10% buffered formalin. The numbers and sizes of polyps and their distributions in the intestine were assessed with a stereoscopic microscope. The colon was opened longitudinally and observed colon tumors were collected. A half part of each colon tumor was stored at −80 °C for PCR analysis, and the other half was fixed with 10% buffered formalin and embedded in paraffin. Paraffin sections were stained with hematoxylin and eosin for histological examination. The remaining intestinal mucosa (non-polyp part) was removed by scraping, and then stored at −80 °C.

### 4.3. Measurement of Mouse Serum Parameter Levels

Serum concentrations of OPN (R&D Systems, Minneapolis, MN, USA) and IL-6 (BioSource International, Inc., Camarillo, CA, USA) were determined by enzyme-linked immunoassays according to the manufacturer’s protocol. The serum levels of TGs were measured using the Fuji Dri-Chem system (Fujifilm, Tokyo, Japan).

### 4.4. Quantitative RT-PCR Analysis

The mRNA expression levels of OPN, MMP-3, MMP-9, MMP-13, MMP-2, MMP-7, Bcl-2, CyclinD1, COX-2, TGF β1, F4/80, CD44, Mest, Snail, Twist, and Vimentin were examined in colorectal tumors (*n* = 5~6 for each group) and non-lesional colorectal mucosa (*n* = 6 for each group). Total RNA was extracted from the tissue samples using TRIZOL^®^ Reagent (Life Technologies, Japan). After RNA purification, aliquots of total RNA (2 µg) were subjected to the RT reaction with oligo-dT and hexamer random primers in a final volume of 20 µL using an iScript ^TM^ cDNA Synthesis Kit (Bio-Rad Lab., Hercules, CA, USA). Quantitative real-time RT-PCR was performed in a final volume of 10 µL with aliquots of cDNA (10 ng) using SsoAdvanced^TM^ Universal SYBR^®^ Green Supermix (Bio-Rad Laboratories, Inc., Hercules, CA) and a PTC-200 DNA engine cycler equipped with a CFD-3220 Opticon 2 detector (MJ Research Inc., St. Bruno, Quebec, Canada) for fluorescence detection. The primers used were selected from the mouse cDNA sequences of GAPDH, OPN, MMP-3, MMP-9, MMP-13, MMP-2, MMP-7, Bcl-2, CyclinD1, COX-2, TGF β1, F4/80, CD44, Mest, Snail, Twist and Vimentin: 5’-primer: 5’-TCAAGAAGGTGGTGAAGCAG-3’, 3’-primer: 5’-TCCACCACCCTGTTGCTGTA-3’ (product size, 203 bp) for GAPDH; 5’-primer: 5’-CTTGCGCCACAGAATGCTG-3’, 3’-primer: 5’-TGACCTCAGTCCATAAGCCA-3’ (product size, 303 bp) for OPN; 5’-primer: 5’-CGTTTCCATCTCTCTCAAGATG-3’, 3’-primer: 5’-GTTAGACTTGGTGGGTACCA-3’ (product size, 99 bp) for MMP-3; 5’-primer: 5’-TGTACCGCTATGGTTACAC-3’, 3’-primer: 5’-CGACACCAAACTGGATGAC-3’ (product size, 372 bp) for MMP-9; 5’-primer: 5’-GATGATGAAACCTGGACAAG-3’, 3’-primer: 5’-GCCAGTGTAGGTATAGATGG-3’ (product size, 138 bp) for MMP-13; 5’-primer: 5’-TCAAGTTCCCCGGCGATGTC-3’, 3’-primer: 5’-AGTTGGCCACATCTGGGTTG-3’ (product size, 225 bp) for MMP-2; 5’-primer: 5’-TGTGGAGTGCCACATGTTGC-3’, 3’-primer: 5’-GTGTTCCCTGGCCCATCAAA-3’ (product size, 266 bp) for MMP-7; 5’-primer: 5’-AGCTGCACCTGACGCCCTTCAC-3’, 3’-primer: 5’-TCCACACACATGACCCCACCGA-3’ (product size, 127 bp) for Bcl-2; 5’-primer: 5’-CCATGGAACACCAGCTCCTG-3’, 3’-primer: 5’-CGGTCCAGGTAGTTCATGGC-3’ (product size, 187 bp) for CyclinD1; 5’-primer: 5’-AATGAGTACCGCAAACGCTT-3’, 3’-primer: 5’-GAGAGACTGAATTGAGGCAG-3’ (product size, 323 bp) for COX-2; 5’-primer: 5’-TTCCTGCTTCTCATGGCCACCC-3’, 3’-primer: 5’-TGCCGCACGCAGCAGTTCTT-3’ (product size, 122 bp) for TGF β1; 5’-primer: 5’-CCTGGACGAATCCTGTGAAG-3’, 3’-primer, 5’-GGTGGGACCACAGAGAGTTG-3’ (product size, 64 bp) for F4/80; 5’-primer: 5’-CTGGATCAGGCATTGATGATG-3’, 3’-primer: 5’-GCCATCCTGGTGGTTGTCTG-3’ (product size, 157 bp) for CD44; 5’-primer: 5’-CTGAGAGTGAGCTGTGGGAC-3’, 3’-primer: 5’-GGCAGCGTTTTCCTGTACAG-3’ (product size, 220 bp) for Mest; 5’-primer: 5’-CATCCGAAGCCACACGCTG-3’, 3’-primer: 5’-CGCAGGTTGGAGCGGTCA-3’ (product size, 256 bp) for Snail; 5’-primer: 5’-GATGGCAAGCTGCAGCTATG-3’, 3’-primer: 5’-CAGCTCCAGAGTCTCTAGAC-3’ (product size, 193 bp) for Twist; 5’-GATTCAGGAACAGCATGTCC-3’, 3’-primer: 5’-CATCCACTTCACAGGTGAG-3’ (product size, 251 bp) for Vimentin. The cycling conditions were as follows: 95 °C for 3 min, 40 cycles of 94 °C for 10 s, 60 °C (GAPDH, OPN, MMP2, Bcl-2, CyclinD1, TGF β1, F4/80, Mest, Vimentin), 55 °C (MMP-3, MMP-9, MMP-13, MMP-7, COX-2, Twist), or 65 °C (CD44, Snail) for 20 s, 72 °C for 20 s, and 79 °C for 2 s. The fluorescence intensity of SYBR Green I was measured at 79 °C at every cycle. To assess the specificity of each primer set, amplicons generated from the PCR reaction were analyzed for melting curves. Finally, the PCR products were analyzed by 2% agarose gel electrophoresis with ethidium bromide staining to confirm the correct sizes. Quantification of OPN, MMP-3, MMP-9, MMP-13, MMP-2, MMP-7, Bcl-2, CyclinD1, COX-2, TGF β1, F4/80, CD44, Mest, Snail, Twist, and Vimentin relative to GAPDH was performed by ΔΔ*C*t method.

### 4.5. Immunohistochemical Staining of Colon Tumors

Paraffin-embedded tissue sections of colorectal tumors were used for immunohistochemical analyses with the avidin-biotin complex immunoperoxidase technique after heating with 10 mM citrate buffer (pH 6.0). As the primary antibodies, polyclonal rabbit anti-MMP-9 immunoglobulin G (IgG) (Chemicon, Temecula, CA, USA) and anti-F4/80 IgG (Santa Cruz Biotechnology, Santa Cruz, CA, USA) were used at 100× and 200× dilution, respectively. As the secondary antibody, biotinylated anti-rabbit IgG (H+L) raised in a goat, affinity purified, (Vector Laboratories Inc., Burlingame, CA, USA) was employed at 200× dilution. Staining was performed using avidin-biotin reagents (Vectastain ABC reagents; Vector Laboratories Inc., Burlingame, CA, USA), 3,3’-diaminobenzidine, and hydrogen peroxide. The sections were counterstained with hematoxylin. As a negative control, duplicate sections were immunostained without exposure to the primary antibody.

### 4.6. Statistical Analysis

The significance of differences in the incidences of colon tumors was analyzed using Fisher’s exact probability test. Other results are expressed as mean ± standard deviation (SD) and statistically analyzed using one-way analysis of variance (ANOVA), followed by Tukey-Kramer multiple comparison post-hoc test. Correlation of serum TG levels or spleen weights with polyp numbers was analyzed by the Pearson correlation test or Spearman’s rank correlation coefficient test. Differences were considered to be statistically significant at *p* < 0.05.

## 5. Conclusions

OPN expression was upregulated in colon tumors in *Apc*-deficient mice and OPN-knockout significantly suppressed tumor development. Though OPN was not essential for tumor formation, it was indicated that OPN is involved in early stage intestinal tumorigenesis in part by upregulation of MMP-3, MMP-9, and MMP-13, and infiltration of macrophages and neutrophils. OPN could be a target for cancer prevention.

## Figures and Tables

**Table 1:** Number of small intestinal polyps in osteopontin (OPN)-deficient Min mice.

Genotype	No. of Animals	Small Intestinal Polyps			
		Duodenum	Middle	Distal	Total
Male					
Min/OPN(+/+)	15	5.1 ± 3.1	40.1 ± 33.9	106.6 ± 66.6	151.9 ± 101.8
Min/OPN(+/−)	29	5.0 ± 3.7	22.8 ± 16.9	72.7 ± 37.6	100.5 ± 53.4
Min/OPN(−/−)	18	6.0 ± 2.5	27.4 ± 21.9	77.1 ± 42.9	110.5 ± 63.9
Female					
Min/OPN(+/+)	10	7.1 ± 4.5	40.1 ± 27.8	106.9 ± 54.4	154.1 ± 85.2
Min/OPN(+/−)	27	4.5 ± 2.3	22.2 ± 17.5 *	65.0 ± 44.7 *	91.7 ± 62.0 *
Min/OPN(−/−)	18	5.2 ± 3.3	28.2 ± 17.5	90.4 ± 44.3	123.7 ± 61.9
Total					
Min/OPN(+/+)	25	5.9 ± 3.8	40.1 ± 31.0	106.7 ± 60.8	152.8 ± 93.6
Min/OPN(+/−)	56	4.8 ± 3.1	22.5 ± 17.0 **	69.0 ± 41.0 **	96.3 ± 57.4 **
Min/OPN(−/−)	36	5.6 ± 2.9	27.8 ± 19.5	83.8 ± 43.5	117.1 ± 62.4

OPN, osteopontin. Data are expressed as mean ± SD. Significant difference from Min/OPN(+/+) mice (* *p* < 0.05, ** *p* < 0.01).

**Table 2:** Incidence and multiplicity of colon tumors in OPN-deficient Min mice.

Genotype	Adenoma		Adenocarcinoma		Total	
	Incidence (%)	Multiplicity ^a^	Incidence (%)	Multiplicity ^a^	Incidence (%)	Multiplicity ^a^
Male						
Min/OPN(+/+)	7/15 (47)	1.0 ± 1.5	8/15 (53)	0.7 ± 0.8	12/15 (80)	1.7 ± 2.0
Min/OPN(+/−)	4/29 (14) *	0.1 ± 0.4 **	13/29 (45)	0.6 ± 0.8	16/29 (55)	0.8 ± 0.8 *
Min/OPN(−/−)	4/18 (22)	0.2 ± 0.4 *	8/18 (44)	0.5 ± 0.6	11/18 (61)	0.7 ± 0.7
Female						
Min/OPN(+/+)	4/10 (40)	0.5 ± 0.7	7/10 (70)	1.0 ± 0.9	8/10 (80)	1.5 ± 1.3
Min/OPN(+/−)	5/27 (19)	0.2 ± 0.4	8/27 (30) *	0.3 ± 0.6 *	11/27 (41) *	0.6 ± 0.7 *
Min/OPN(−/−)	6/18 (33)	0.4 ± 0.7	5/18 (28) *	0.3 ± 0.6 *	7/18 (39) *	0.8 ± 1.2
Total						
Min/OPN(+/+)	11/25 (44)	0.8 ± 1.2	15/25 (60)	0.8 ± 0.9	20/25 (80)	1.6 ± 1.7
Min/OPN(+/−)	9/56 (16) **	0.2 ± 0.4 **	21/56 (38)	0.5 ± 0.7	27/56 (48) **	0.6 ± 0.8 **
Min/OPN(−/−)	10/36 (28)	0.3 ± 0.6 *	13/36 (36)	0.4 ± 0.6	18/36 (50) *	0.8 ± 0.9 **

OPN, osteopontin. ^a^ Data are expressed as mean ± SD. Significant difference from Min/OPN(+/+) mice (* *p* < 0.05, ** *p* < 0.01).

**Table 3:** Effects of OPN deficiency on serum levels of OPN, interleukin (IL)-6, and triglycerides (TGs).

Genotype	Serum OPN (ng/mL)	Serum IL-6 (pg/mL)	Serum TGs (mg/dL)
Min/OPN(+/+)	456.7 ± 144.7 ^a^	50.5 ± 16.7 ^a^	464 ± 383 ^a^
Min/OPN(+/−)	250.4 ± 73.4 ^b^	54.8 ± 27.7 ^a^	401 ± 454 ^a^
Min/OPN(−/−)	0 ^d^	22.8 ± 28.3 ^b^	360 ± 349 ^a^
OPN(+/+)	414.5 ± 192.9 ^a^	0 ^b^	94 ± 20 ^b^
OPN(+/−)	182.3 ± 88.2 ^c^	5.0 ± 14.6 ^b^	91 ± 25 ^b^
OPN(−/−)	0 ^d^	0 ^b^	97 ± 61 ^b^

Data are means ± SD. Values that do not share a common superscript are significantly different at *p* < 0.01, except serum IL-6 levels in Min/OPN(+/+) and Min/OPN(+/−) (*p* < 0.05).

## Supplementary Materials

The following is available online at www.mdpi.com/1422-0067/18/5/1058/s1, Figure S1: Effects of OPN deficiency on body and spleen weights, Figure S2: A macroscopic view of the colorectum of (a) male Min/OPN(+/+), (b) male Min/OPN(+/−), (c) male Min/OPN(−/−), (d) female Min/OPN(+/+), (e) female Min/OPN(+/−), and (f) female Min/OPN(−/−).
