# Supplementary material for: Osteopontin Deficiency Suppresses Intestinal Tumor Development in Apc-Deficient Min Mice
Source: Int J Mol Sci. 2017 May 14;18(5):1058. doi: 10.3390/ijms18051058 (PMC5454970; doi:10.3390/ijms18051058)
Supplement: Supplementary file 1 [file ijms-18-01058-s001.pdf]

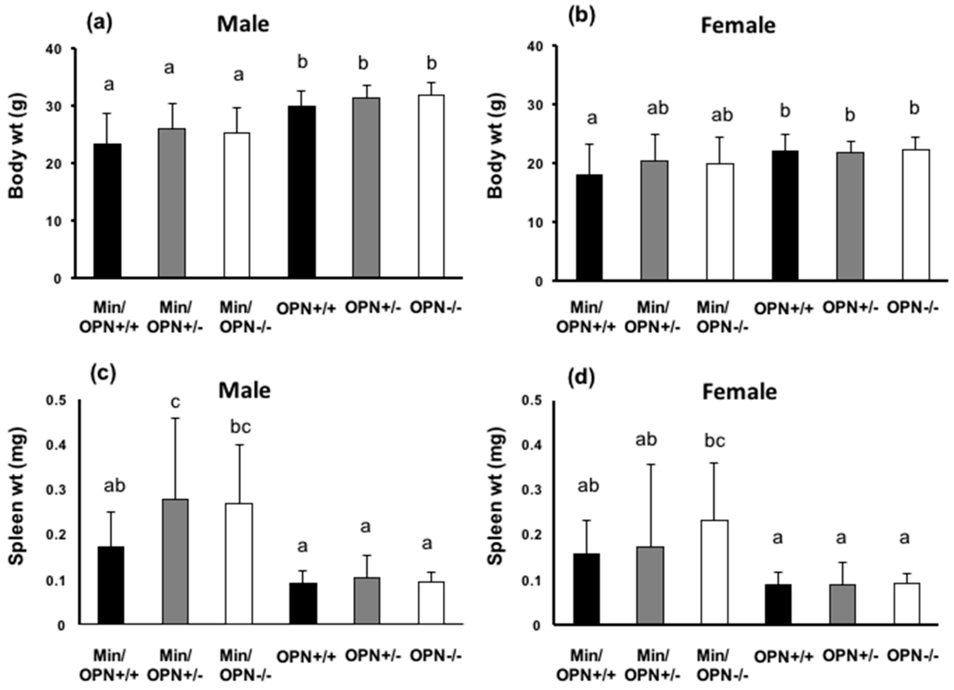

Figure S1. Effects of OPN deficiency on body and spleen weights. Values for body weight of (a) male and (b) female mice. Values for spleen weight of (c) male and (d) female mice. Data are means ± SD. Values that do not share a common superscript are significantly different at *P*<0.05.

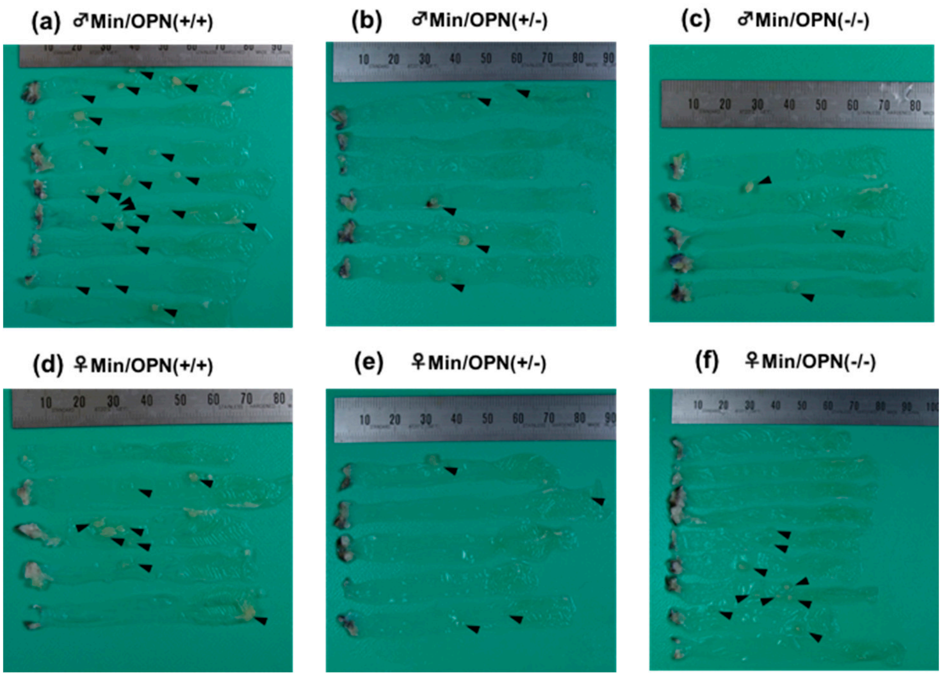

Figure S2. A macroscopic view of the colorectum of (a) male Min/OPN(+/+) , (b) male Min/OPN(+/-), (c) male Min/OPN(-/-), (d) female Min/OPN(+/+), (e) female Min/OPN(+/-), and (f) female Min/OPN(-/-).
